# Associations between meeting 24h movement behavior guidelines and cognition, gray matter volume, and academic performance in children and adolescents: a systematic review

**DOI:** 10.1186/s13690-024-01493-0

**Published:** 2025-01-10

**Authors:** Bo Liu, Peng Shi, Teng Jin, Xiaosu Feng

**Affiliations:** 1 School of Athletic Performance, Shanghai University of Sport, Shanghai, 200438 China; 2 School of Physical Education, Shanghai University of Sport, Shanghai, 200438 China; 3 School of Physical Education, Shandong University of Technology, Zibo, 255000 China; 4 School of Physical Education, Liaoning Normal University, Dalian, 116029 China

**Keywords:** 24-hour movement behaviors, Cognition, Gray matter volume, Academic performance, Children and adolescents

## Abstract

**Background:**

24-h movement behaviors have a close relationship with children and adolescents' cognition, gray matter volume, and academic performance. This systematic review aims to precisely explore the associations between meeting different combinations of guidelines and the aforementioned indicators, in order to better serve public health policy.

**Methods:**

Computer retrieval was conducted on CNKI, Web of Science, PubMed, SPORT Discus and Cochrane library databases. The screening and data extraction processes were conducted by two researchers. This study used the Joanna Briggs Institute checklist for methodological quality assessment and the Grading of Recommendations Assessment, Development, and Evaluation system for the evaluation of the level of evidence. Descriptive statistical analysis is performed using frequency and percentage on the extracted data and key findings, primarily to assess the consistency of the positive benefits associated with meeting different guidelines and outcome variables.

**Results:**

A total of 10 studies were included (with 16 correlation analyses conducted), involving 51,566 children and adolescents aged between 4.2 and 15.9 years old. The included studies generally agreed upon the following associations: adherence to the screen time (ST) guidelines is positively linked to fluid intelligence; adherence to the sleep duration (SD) guidelines is positively linked to literacy; adherence to both ST and SD guidelines is associated with increased fluid intelligence and gray matter volume; and overall adherence to all guidelines is positively correlated with fluid intelligence. The included studies reported low certainty of evidence. Additionally, the included studies have provided clear evidence, but some studies did not strictly control confounding factors, and it is also unclear whether there is a larger effect size, hence the level of evidence is relatively low.

**Conclusion:**

There are varying degrees of associations between different combinations of guidelines and cognition, gray matter volume, and academic performance, but further research is needed to confirm these findings, especially the relatively limited role of meeting physical activity guidelines.

**Supplementary Information:**

The online version contains supplementary material available at 10.1186/s13690-024-01493-0.

**Table:** 

Text box 1. Contributions to the literature
• Systematic review of the association of meeting 24-h movement behavioral guidelines with cognition, gray matter volume, and academic performance in children and adolescents provides evidence for public health policy development.
• The associations of meeting physical activity guidelines with cognition, gray matter volume, and academic performance reported low certainty of evidence and require further validation.
• Associations between meeting different combinations of guidelines and cognition, gray matter volume, and academic performance exist to varying degrees.

## Introduction

Cognition is the process of acquiring and processing information, which serves as the foundation for understanding the world, communicating with others, and making daily decisions [1]. The gray matter, composed of neuronal cell bodies, is extensively distributed in the cerebral cortex and deep brain areas, forming brain regions related to motor control, perception, decision-making, and self-control [2]. In other words, there is a close connection between the volume of gray matter and cognitive functions [3]. The period of childhood and adolescence is a critical stage for brain and cognitive development, where effective stimulation can promote brain plasticity [4], leading to positive effects on learning efficiency, personality shaping, and social skills [5, 6]. In addition, academic performance is a comprehensive reflection of brain structure and function in learning situation tasks [7], and is a primary indicator for measuring the level of education in children and adolescents. It is not only the main goal of students' development but also the target pursued by stakeholders such as teachers and parents. Therefore, promoting cognition and enhancing academic performance among children and adolescents are key topics of interest for researchers.

The cognitive and academic performance of children and adolescents is closely related to their lifestyle and habits [8, 9]. Physical activity (PA), sedentary behavior (SB), and sleep duration (SD) are the main lifestyle components of children and adolescents [10]. Especially in the context of technological advancements and social development, children and adolescents globally are facing issues such as insufficient PA, excessive SB, and unreasonable SD [11–13]. Such unhealthy activity patterns can lead to a decline in brain and cognitive functions, which in turn can impair academic performance [14]. Based on this, numerous studies have focused on exploring the association between PA, SB, and SD with the cognitive and academic performance of children and adolescents, aiming to provide quantitative evidence for subsequent studies to promote cognitive benefits and academic performance by improving their lifestyle.

In early studies on children and adolescents, researchers mainly focused on exploring the independent associations of PA, SB, and SD with cognitive and academic performance. For example, numerous studies [15–17] have found that PA time is significantly positively associated with cognitive tasks such as intelligence and inhibitory control, as well as academic performance, and cognitive function plays a mediating role in the association between PA and academic performance. Additionally, screen-based SB time has been found to have a significant negative correlation with executive function task performance among children and adolescents [18]. Insufficient SD has also been shown to have a significant negative correlation with cognitive and academic performance [19, 20]. In addition, beyond independent associations, some studies have gradually paid attention to the combined associations of two variables among PA, SB, and SD with cognitive benefits. For instance, Cui et al. [21] found that SB and sleep disorders have a negative multiplicative interaction effect on the prevalence of cognitive dysfunction among the elderly. However, unfortunately, such interaction studies are still rare among children and adolescents. In other outcome variables, some researchers [22–25] have found that a combination of higher moderate-to-vigorous-intensity physical activity (MVPA) and lower SB time is associated with body fat percentage, muscle mass, bone density, and physical posture health among children and adolescents.

From the perspective of activity behavior, PA, SB, and SD constitute 24h of activity, and these behaviors are interdependent and covariant, and a change in one behavior will inevitably lead to a change in the other behaviors [26]. Therefore, many researchers [27–29] have pointed out that only by having appropriate amounts of PA, high-quality SB, and sufficient SD, while maintaining a relative balance, can one achieve more comprehensive health benefits. Based on this, some studies [30–32] have proposed that guidelines incorporating 24h of activity behavior are more reasonable compared to standalone PA or SB guidelines. Subsequently, countries such as Canada and Australia have developed 24h movement behavior guidelines for children and adolescents, recommending at least 60 min or more of MVPA per day, less than 2h of screen time (ST), and 9–11 h of SD for children aged 5–13 and 8–10 h of SD for adolescents aged 14–17 [28, 33]. These guidelines provide a basis for guiding and maintaining a healthy lifestyle among children and adolescents, attracting significant attention from researchers in fields such as public health and physical education.

Researchers have gradually paid attention to the association between 24h movement behaviors and cognition and academic performance among children and adolescents, conducting relevant empirical studies. However, the results of these studies are inconsistent. For instance, Zeng et al. [34] found that children and adolescents who meet the 24h movement behavior guidelines exhibit higher performance on executive function tasks. However, McNeill et al. [35] did not find an association between the two. Additionally, Lien et al. [36] found that children and adolescents who meet the 24h movement behavior guidelines have higher academic performance, but studies by Tapia-Serrano et al. [37] and Howie et al. [38] do not support this finding. Therefore, some studies [39–44] are looking forward to conducting a systematic review to collect, synthesize, and analyze existing studies, providing a comprehensive, objective, and scientific assessment of this specific issue, in the hope of providing a scientific basis for policy-making and subsequent research. However, the aforementioned systematic reviews still have some limitations. First, previous study [41] has explored the association between 24h movement behaviors and health outcome indicators across the entire life cycle, rather than specifically investigating the children and adolescents, and the pertinence of the research results is still insufficient. Secondly, considering the limitations of the entire life cycle population, most studies [39, 40, 42–44] have focused on exploring the association between 24h movement behaviors and overweight/obesity, mental health, motor development, and cognitive development in children and adolescents, achieving positive outcomes. However, previous studies [39, 42, 43] have only included one original study on cognitive development, which may lead to potential bias risks, greatly reducing the accuracy of the results. Finally, this study has not yet found a systematic review exploring the association between 24h movement behaviors and academic performance.

Based on this, the present study aims to explore associations between meeting 24h movement behavior guidelines and cognition, gray matter volume, and academic performance in children and adolescents using a systematic review approach. This study aims to enrich research in the field of 24h movement behaviors among children and adolescents, providing evidence support for enhancing their cognition and academic performance and informing the development of relevant policies.

## Methods

This study strictly followed the Preferred Reporting Items for Systematic Review and Meta-Analyses (PRISMA) [45] for the steps of search, screening, assessment, data extraction, and consolidation of results. The complete PRISMA 2020 checklist can be found in the supplementary material 1. This systematic review was registered (CRD 42024527063) in the International Prospective Register of Systematic Reviews (PRO SPERO).

### Search strategies

This study was conducted by a single researcher using a strategy of searching both Chinese and English subject terms in various databases, including CNKI, WOS, PubMed, SPORT Discus, and Cochrane Library. The search terms included: (1) children OR adolescent OR youth OR teenager OR preschool OR juvenile OR pupil OR primary OR elementary OR high school OR junior school OR senior school; AND (2) 24-h OR 24-h movement; AND (3) cognitive OR executive function OR attention OR self-control OR gray matter OR academic performance. Boolean operators "AND" and "OR" were used to connect the search terms for the retrieval process. This study is based on the precise retrieval methods in advanced search, using Boolean operators to connect the subject terms in order to retrieve relevant subject literature from the database. Additionally, for CNKI, this study uses the asterisk "*" and the plus sign " + " to concatenate strings. The asterisk "*" is used to connect different types of search terms, consistent with the meaning of "AND"; the plus sign " + " is used to connect the same type of search terms, consistent with the meaning of "OR". If a specific database has not yet retrieved the corresponding articles through title search, then a more relaxed full-text search will be used for the retrieval. The specific retrieval strategy is detailed in Supplementary Material 2. The search was conducted from the establishment of the databases up to July 2024.

### Selection criteria

Inclusion criteria: (1) The participants are healthy children and adolescents without special diseases; (2) The independent variable is 24-h movement behaviors; (3) The dependent variable is one, multiple, or all of cognitive function, gray matter volume, and academic performance; (4) The original study is a cross-sectional study. Exclusion criteria: (1) The participants are atypical groups of children and adolescents; (2) The participants are athletes in the child and adolescent stage; (3) Studies focusing on a single variable or pairwise combinations of PA, ST, and SD; (4) Reviews, abstracts, commentaries, editorials, etc.; (5) In cases of duplicate publications, only the study with relatively higher research quality is included. The screening process will be conducted independently by two researchers. The two researchers will then cross-check the selected literature. If there are any controversial articles, a group discussion will be held to make a joint decision.

### Data codes

The information extracted from the literature encompasses: (1) bibliographic details, specifically the first author and the year of publication; (2) characteristics of the study participants, including the sample size, age range, percentage of female participants, and nationality; (3) exposure factors, such as PA, ST, and SD, along with the respective measurement tools employed; (4) outcome variables, such as cognitive function, gray matter volume, and academic performance, together with the measurement techniques used; (5) the statistical methods used in the original study; and (5) confounding factors that could potentially influence the study results. This study has entered the aforementioned extracted data into the Excel 2010 software, and the detailed results of the data extraction can be seen in Table 1. In cases where the same study investigates different age groups or genders separately, the information will be extracted and coded accordingly for each subpopulation. This process will be carried out independently by two researchers, followed by a cross-verification of the extracted data. Any discrepancies or controversies encountered during this process will be resolved through a group discussion among the researchers to ensure a consensus is reached.

**Table 1:** Basic information on the included studies

Included studies	Participants (*n*, age, F%, nationality)	Exposure factors (measurement tools)	Outcome variables (measurement tools)	Main statistical techniques	Confounders
Hinkley et al. [46], 2020	*n*=498 8~9 years N/A Australia	PA (accelerometer) ST (parent-reported) SD (parent-reported)	Reading (NAPLAN) Writing (NAPLAN) Spelling (NAPLAN) Numeracy (NAPLAN) Language (NAPLAN)	Linear regression model	age, gender, maternal education, BMI
Howie et al. [38], 2020	*n*=934 11~18 years N/A Australia	PA (ANPARC) ST (TechU-Q) SD (CRSP)	Overall academic performance (grade point average) Math Performance (Math score) English Performance (English score)	Linear regression model	gender, school grade
Lien et al. [36], 2020	(1) *n*=10160 15.1±1.8 years 49.1% girls Canada (2) *n*=3196 12.7±0.8 years 50.7% girls Canada (3)* n*=6964 15.9±1.2 years 51.0% girls Canada	PA, ST, and SD (self-reported)	Overall academic performance (coded mean score)	Linear regression model	age, gender, ethnicity, SES, BMI, addictive substance use
Tapia-Serrano et al. [37], 2022	(1)* n*=1227 11~16 years 44.2% girls Spanish (2)* n*=712 11~16 years 0.0% girls Spanish (3)* n*=565 11~16 years 100.0% girls Spanish	PA (PAQ-A) ST (YLSBQ) SD (self-reported)	Overall academic performance (average scores in Spanish, English and Math)	ANCOVA	age, gender, SES, BMI, CRF
Walsh et al. [47], 2018	*n*=4520 9~10 years 52.0% girls USA	PA (YRBS) ST (YSTS) SD (PSDSC)	Crystal intelligence (NIH Toolbox) Fluid intelligence (NIH Toolbox) Global cognition (NIH Toolbox)	Random intercepts model	gender, ethnicity, BMI, grades, household income, educational attainment of parents, multilingual status, adolescent status, brain injury status
Fung et al. [48], 2023	(1)* n*=10574 9~11years 48.0% girls USA (2)* n*=9273 11~14 years 47.7% girls USA	PA, ST, and SD (self-reported and parent-reported)	Crystal intelligence (NIH Toolbox) Fluid intelligence (NIH Toolbox) Global cognition (NIH Toolbox) GMVs (Brain scans)	Linear mixed effects model	age, gender, ethnicity, household income, educational attainment of parents
Zeng et al. [34], 2022	*n*=376 7~12 years 48.1% girls China	PA (accelerometer) ST (self-reported) SD (self-reported)	Executive function (WCST)	Generalized linear mixed model	age, gender, BMI, household income, educational attainment of parents, IQ
McNeill et al. [35], 2020	*n*=247 4.2±0.6 years N/A Australia	PA (accelerometer) ST (parent-reported) SD (parent-reported)	Visuo-spatial working memory (Mr Ant) Verbal working memory (Not This) Inhibition (GO NOGO) Switching (Card Sort)	Linear regression model	age, gender, SES, educational attainment of parents, education expenditures
McGowan et al. [49], 2023	*n*=123 4.9±0.7 years 52.8% USA	PA (YRBS) ST (YSTS) SD (BISQ)	Self-regulation (Off-task behavior)	Linear mixed model	age, gender, BMI
Watson et al. [50], 2022	(1)* n*=1270 12.0±0.4 years 48.0% Australia	PA, ST, SD (MARCA)	Literacy (National Assessment Program) Numeracy (National Assessment Program)	ANCOVA	age, gender, SES, educational attainment of parents, household income, occupations of parents, stage of pubertal development
	(2)* n*=927 12.0±0.4 years 48.0% Australia	PA (accelerometer) ST (accelerometer) SD (MARCA)			

Notes: *n* represents the sample size; F% stands for the proportion of females; N/A indicates unknown or not applicable; ANCOVA refers to one-way covariance analysis; YRBS refers to the Youth Risk Behavior Survey; PSDSC stands for the Parent Sleep Disturbance Scale for Children; GMVs represent gray matter volumes; PAQ-A stands for the Physical Activity Questionnaire for Adolescents; YLSBQ refers to the Youth Leisure-Time Sedentary Behavior Questionnaire; YSTS stands for the Youth Screen Time Survey; CRSP refers to the Children’s Report of Sleep Patterns; ANPARC stands for the Australian National Physical Activity Report Card; TechU-Q stands for the Technology Use Questionnaire; BISQ refers to Brief Infant Sleep Questionnaire; MARCA refers to Multimedia Activity Recall for Children and Adolescents; WCST refers to the Wisconsin Card Sorting Test; NAPLAN refers to the standardized National Assessment Program–Literacy and Numeracy; BMI stands for body mass index; SES stands for socioeconomic status; CRF refers to cardiorespiratory fitness.

### Quality assessment

In this study, the quality of the included cross-sectional studies was evaluated using the Joanna Briggs Institute (JBI) checklist developed by the Joanna Briggs Institute for Evidence-Based Nursing in Australia [51]. This tool comprises a total of 10 items, and evaluators are required to assign a score of 0, 1, or 2 to each item. A score of 0 indicates that the requirement is not met; a score of 1 indicates that the item is mentioned but not described in detail; and a score of 2 indicates that the item is described in a detailed, comprehensive, and accurate manner. Additionally, this study employs the Grading of Recommendations Assessment, Development, and Evaluation (GRADE) system to rate the quality of evidence for each outcome variable [52]. Within the GRADE system, there are five factors that can downgrade the quality of evidence: study limitations, imprecision, inconsistency of results, indirect evidence, and potential publication bias. Moreover, there are three factors that can upgrade the quality of evidence: a large effect size, dose-response relationship, and the reduction of confounding factors to a minimum [52]. Since all studies included in this research are observational, the grading process starts from a lower level and then proceeds to upgrade the study results accordingly. Two researchers independently conducted the literature quality evaluation, and they cross-checked the results after the evaluation. In cases of disputes or controversies, a group discussion was held to reach a consensus.

### Data analysis

This study primarily employs frequency and percentage for descriptive statistical analysis of the extracted data and key findings. Firstly, the study provides descriptive statistics for the main characteristics of the participants (age, proportion of females, nationality), the measurement tools used for 24-h movement behavior, the main statistical techniques employed in the study, and the relevant confounding factors. Secondly, the study organizes whether the original research meets the 24-h movement behavior guidelines and the relationship with cognition, gray matter volume, and academic performance, extracts relevant research evidence, and uses frequency analysis to statistically analyze the evidence with positive benefits. Finally, the results of the aforementioned data analysis methods are all presented in tables.

## Results

### Literature screening results

**Fig. 1:**
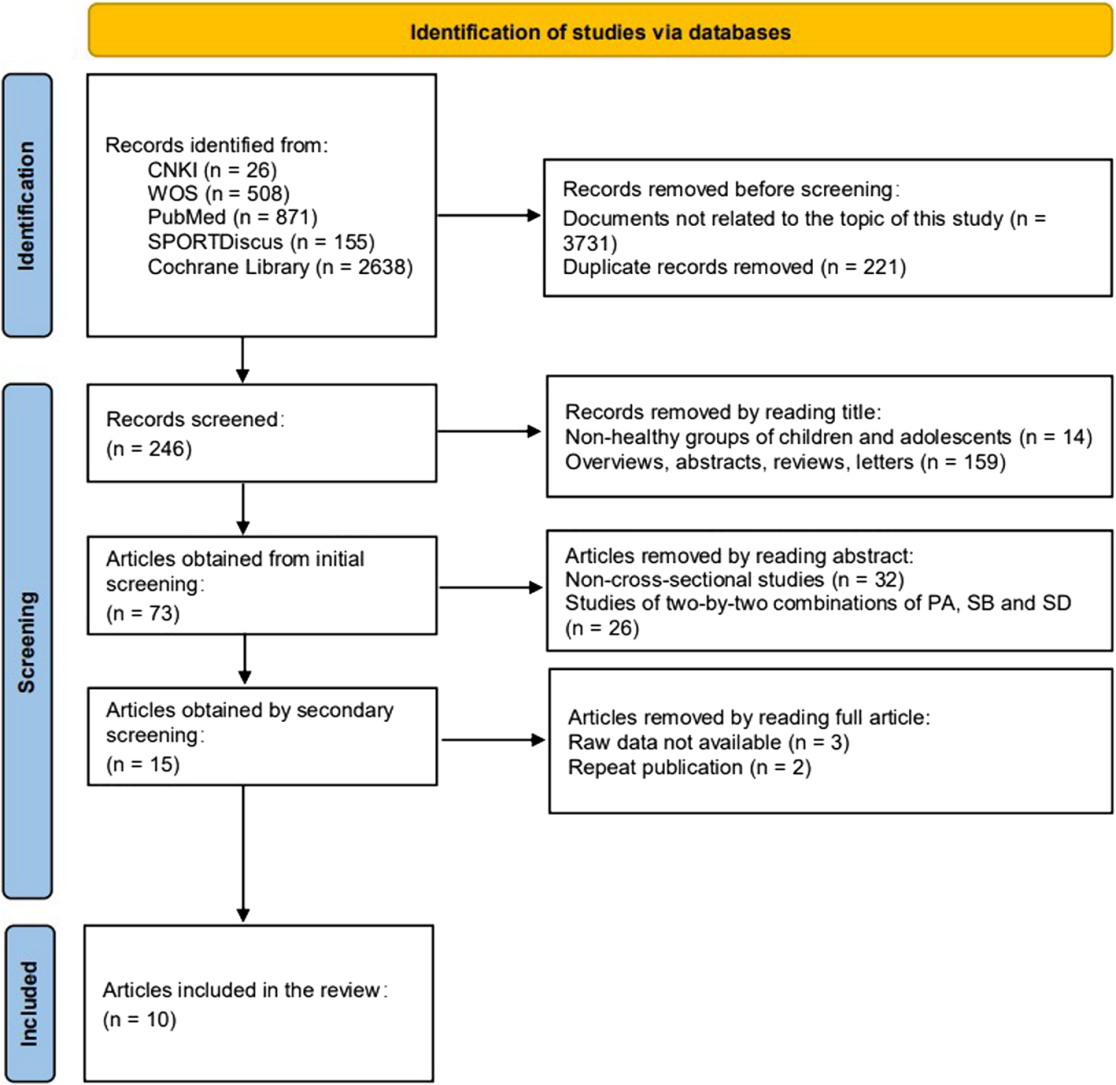
Literature screening process

A total of 4198 documents were retrieved for this study. After excluding 3731 documents and 221 duplicates that were not related to the topic of this study, 246 documents were obtained. Through literature screening, a total of 10 documents were included. The literature screening process is shown in Fig. 1.

### Basic information on the included studies

#### Bibliographic information and participants information

The publication years of the studies included in the research range from 2018 to 2023, comprising 1 study from 2018, 6 studies from 2020, 6 studies from 2022, and 3 studies from 2023. The total sample size of the included studies is 51,566, with the sample size ranging from 123 [49] to 10,574 [48]. The average age of the participants in the studies ranges from 4.2 to 15.9 years old, including 2 studies [35, 49] on preschool children, 6 studies [34, 36, 46–48, 50] on children, 1 study [36] on adolescents, and 2 studies [37, 38] on both children and adolescents. There are 4 studies [36, 37, 47, 49] where the proportion of female children is greater than 50%, 5 studies [34, 36, 37, 48, 50] where it is less than 50%, and 3 studies [35, 38, 46] with an uncertain proportion of female children. Participants come from Australia [35, 38, 46, 50], the United States [47–49], Canada [36], Spain [37], and China [34].

#### Measurement of variables

Included studies primarily adopted subjective and objective measurement methods to collect 24-h movement behavior data from the participants. For PA, 7 studies [36–38, 47–50] used questionnaires for self-assessment or parent-assessment with high reliability and validity, and 4 studies [34, 35, 46, 50] used accelerometers for objective measurement. For ST, 10 studies [34–38, 46–50] used questionnaires for self-assessment or parent-assessment with high reliability and validity, and 1 study [50] used an accelerometer for objective measurement. For SD, all studies used questionnaires for self-assessment or parent-assessment with high reliability and validity.

In the included studies, 5 studies [34, 35, 47–49] explored the association between adherence to the 24-h movement behavior guidelines and cognition, 1 study [48] investigated the association between adherence to the 24-h movement behavior guidelines and gray matter volume, and 5 studies [36–38, 46, 50] examined the association between adherence to the 24-h movement behavior guidelines and academic performance. The outcome variables of Walsh et al. [47] and Fung et al. [48] were global cognition, crystallized intelligence, and fluid intelligence, while the outcome variables of Zeng [34], McNeill et al. [35], and McGowan et al. [49] involved various dimensions of executive function. Among them, Zeng [34] and others used the Wisconsin Card Sorting Test (WCST) to measure participants' planning and problem-solving abilities; McNeill et al. [35] and McGowan et al. [49] used GO/NOGO and Off-task behavior to measure inhibitory control, respectively; McNeill et al. [35] used the Card Sort to assess task switching; in addition, McNeill et al. [35] also assessed visual-spatial working memory and verbal working memory. Hinkley et al. [46], Howie et al. [38], and Watson et al. [50] assessed literacy and numeracy, among which Hinkley et al. [46] used reading, writing, spelling, and language to evaluate literacy achievements, and therefore, they were statistically calculated separately in subsequent calculations. Finally, 3 studies [36–38] explored the association between adherence to the 24-h movement behavior guidelines and overall academic performance.

#### Main statistical techniques and confounding factors

The studies included in the review employed various statistical techniques for analysis. Four studies [35, 36, 38, 46] used linear regression models for analysis; two studies [37, 50] used one-way covariance analysis (ANCOVA) for analysis; three studies [34, 48, 49] used linear mixed models for analysis; and one study [47] used a random intercepts model for analysis. The inconsistency in statistical techniques also posed difficulties in making horizontal comparisons of effect sizes.

Confounding factors include both individual and family levels. At the individual level, confounding factors include age, gender, race, and body mass index (BMI). At the family level, confounding factors include socioeconomic status (SES), family income, and parental education level. In addition, other individual-level confounding factors include school grade, addictive substance use, cardiorespiratory fitness (CRF), multilingual status, stage of pubertal development, brain injury, and IQ. Other family-level confounding factors include maternal education level, educational support, and parental occupation.

**Fig. 2:**
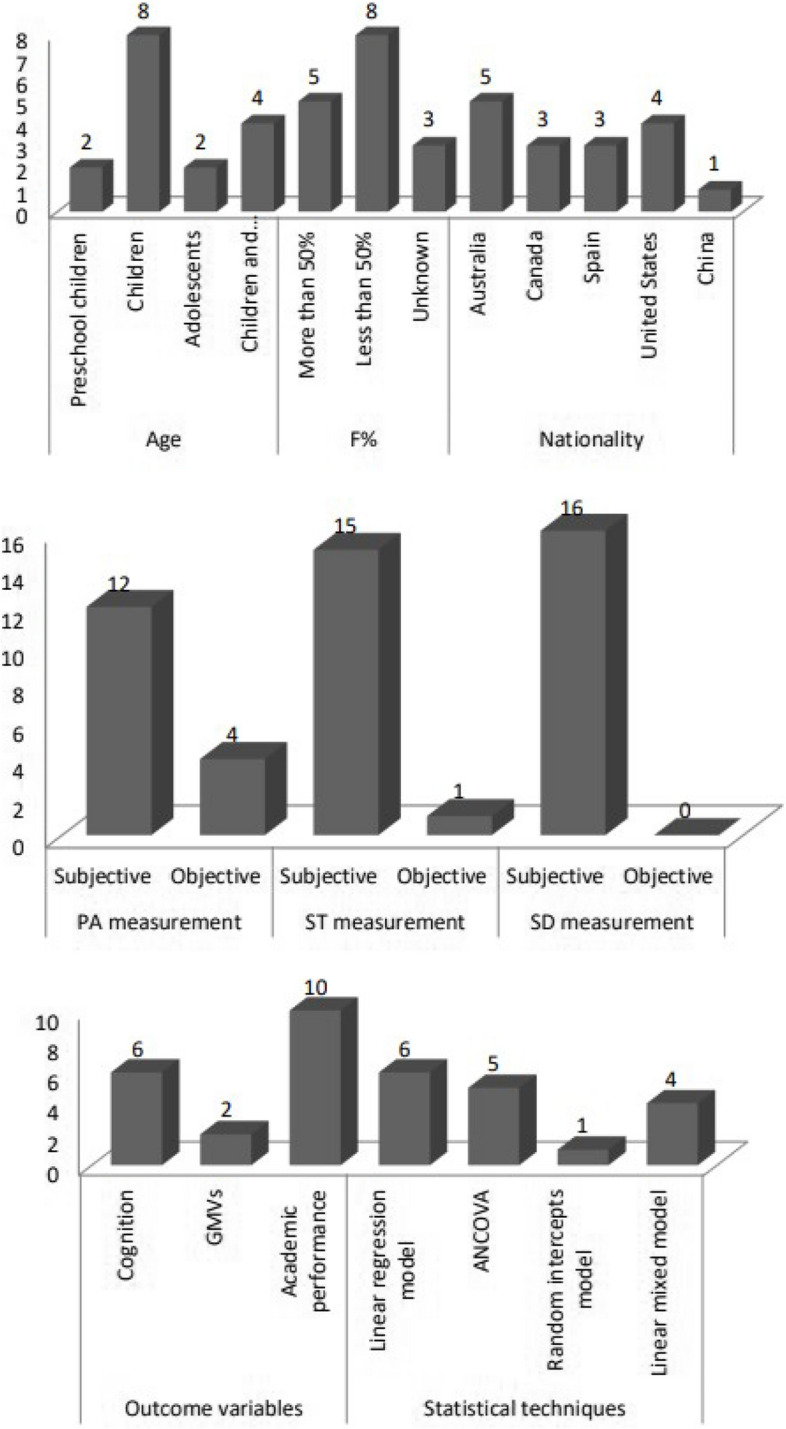
Classification of main characteristics of included studies. Notes: The numbers on the bar chart indicate the number of correlation analyses included

Detailed information about the included studies can be found in Table 1.; the age range of participants, the proportion of females, nationality, as well as the measurement methods of 24-h movement behaviors, the main outcome variables, and the main statistical techniques used are detailed in Fig. 2.

### Quality assessment results

**Fig. 3:**
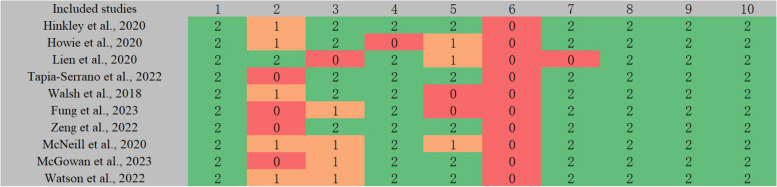
Quality assessment results of included studies. Notes: Gray 1 indicates "Whether the research purpose of the study is clear? Whether the basis for the research question is sufficient?"; Gray 2 indicates "How were the research participants selected?"; Gray 3 indicates "Whether the inclusion and exclusion criteria for the samples are clearly described?"; Gray 4 indicates "Whether the characteristics of the samples are clearly described?"; Gray 5 indicates "Does the tool used for data collection have reliability and validity?"; Gray 6 indicates "What measures were taken to verify the authenticity of the data?"; Gray 7 indicates "Whether ethical issues were taken into account?"; Gray 8 indicates "Whether the statistical methods used are correct?"; Gray 9 indicates "Whether the presentation of the research results is appropriate and accurate?"; and Gray 10 indicates "Whether the research value is clearly explained?". Red 0 indicates "Does not meet the requirements"; Orange 1 means "Mentioned, but not described in detail"; Green 2 signifies "Detailed, comprehensive, and accurate description"

The quality assessment results of the included studies (Fig. 3) reveal that all study comprehensively, thoroughly, and accurately provided the research objectives and rationales, employed correct statistical methods, and appropriately and precisely presented the research findings and their value. The majority of the articles strictly reported on ethical issues and clearly described the characteristics of the samples. A minority of the literature reported on the selection strategies and screening criteria for subjects, as well as the reliability and validity of the data collection tools. However, none of the articles reported measures to verify the authenticity of the data.

### Outcomes

#### Cognitive function

According to Table 2, it can be observed that adherence to different combinations of guidelines is associated with various cognitive indicators to varying degrees. Firstly, compliance with the PA guidelines is positively associated with executive function measured by WCST [34]. Secondly, there are inconsistencies in the associations between compliance with the ST guidelines, SD guidelines, PA and ST guidelines, PA and SD guidelines, ST and SD guidelines, and compliance with all guidelines, especially in terms of global cognition and inhibitory function. Walsh et al. [47] and Fung et al. [48] found that compliance with the ST guidelines, ST and SD guidelines, and all guidelines is positively associated with global cognition, crystallized intelligence, and fluid intelligence in 9 to 11-year-old children, but this finding does not apply to 11 to 14-year-old children [48]. Fung et al. [48] also found that compliance with the SD guidelines is positively associated with crystallized intelligence in 9 to 11-year-old children, which is inconsistent with the research by Walsh et al. [47] on 9 to 10-year-old children and by Fung et al. [48] on 11 to 14-year-old children. In addition, McGowan et al. [49] found that compliance with the ST guidelines, PA and ST guidelines, and PA and SD guidelines is positively associated with inhibitory function in preschool children measured by the Off-task behavior task, while McNeill et al. [35] showed that there is no significant association between compliance with the 24-h movement behavior guidelines and inhibitory function in preschool children measured by the GO/NOGO task. Lastly, compliance with the SD guidelines, PA and SD guidelines, PA and ST guidelines, ST and SD guidelines, and all guidelines is positively associated with executive function measured by WCST [34]. In addition, compliance with the PA and SD guidelines is positively associated with shifting function and verbal working memory, while the association with visuospatial working memory is not significant [35].

**Table 2:** Summarize the beneficial outcomes related to the relationship between adherence to the 24-h movement behavior guidelines and cognition

Variables	PA	ST	SD	PA+ST	PA+SD	ST+SD	PA+ST+SD
Crystallized intelligence		Fung et al., 2023(1); Walsh et al., 2018	Fung et al., 2023(1)			Fung et al., 2023(1); Walsh et al., 2018	Fung et al., 2023(1); Walsh et al., 2018
Fluid intelligence		Fung et al., 2023(1); Walsh et al., 2018				Fung et al., 2023(1); Walsh et al., 2018	Fung et al., 2023(1); Walsh et al., 2018
Global cognition		Fung et al., 2023(1); Walsh et al., 2018				Fung et al., 2023(1); Walsh et al., 2018	Fung et al., 2023(1); Walsh et al., 2018
Executive function (WCST)	Zeng et al.,2022		Zeng et al., 2022	Zeng et al., 2022	Zeng et al., 2022	Zeng et al., 2022	Zeng et al., 2022
Inhibitory function		McGowan et al., 2023		McGowan et al., 2023	McGowan et al., 2023		
Switching function					McNeill et al., 2020		
Visual-spatial working memory							
Language working memory					McNeill et al., 2020		

#### Gray matter volume

According to Table 3, it can be seen that adherence to the ST and SD guidelines is significantly positively associated with gray matter volume [48]. In addition, adherence to the ST guidelines is positively associated with gray matter volume in children aged 9 to 11, but this finding does not apply to children aged 11 to 14; adherence to all guidelines is positively associated with gray matter volume in children aged 11 to 14, and similarly, this finding does not apply to children aged 9 to 11 [48]. However, the associations between adherence to the PA guidelines, SD guidelines, PA and ST guidelines, and PA and SD guidelines with gray matter volume are not significant [48].

**Table 3:** Summarize the beneficial outcomes related to the relationship between adherence to the 24-h movement behavior guidelines and gray matter volume

Variable	PA	ST	SD	PA+ST	PA+SD	ST+SD	PA+ST+SD
GMVs		Fung et al., 2023(1)				Fung et al., 2023(1); Fung et al., 2023(2)	Fung et al., 2023(2)

#### Academic performance

According to Table 4, it can be observed that adherence to different combinations of guidelines is associated with academic performance to varying degrees. Firstly, only one study each showed that meeting the PA guidelines [37] and meeting the ST guidelines [36] are positively associated with overall academic performance; only Watson et al. [50] showed that meeting both PA and ST guidelines are significantly positively associated with numeracy. Secondly, almost all studies [36–38, 46, 50] support the significant positive association between meeting the SD guidelines and overall academic performance, literacy, and numeracy. Finally, the association between meeting PA and SD guidelines, ST and SD guidelines, and all guidelines with academic performance is still inconsistent. Tapia-Serrano et al. [37] found that meeting PA and SD guidelines are significantly positively associated with overall academic performance, but this finding was not further reflected in the study by Lien et al. [36]. Similarly, Lien et al. [36] found that meeting ST and SD guidelines and all guidelines are significantly positively associated with overall academic performance, but this finding has not yet been confirmed in the study by Tapia-Serrano et al. [37]. In addition, only Watson et al. [50] found a positive association between meeting ST and SD guidelines and all guidelines with literacy and numeracy, as well as a positive association between meeting PA and SD guidelines with numeracy, but this finding has not been further confirmed in other studies.

**Table 4:** Summarize the beneficial outcomes related to the relationship between adherence to the 24-h movement behavior guidelines and academic performance

Variables	PA	ST	SD	PA+ST	PA+SD	ST+SD	PA+ST+SD
Overall academic performance	Tapia-Serrano et al., 2022(1)	Lien et al., 2020(2)	Tapia-Serrano et al., 2022(1); Tapia-Serrano et al., 2022(2); Tapia-Serrano et al., 2022(3); Lien et al., 2020(2); Howie et al., 2020		Tapia-Serrano et al., 2022(1); Tapia-Serrano et al., 2022(2); Tapia-Serrano et al., 2022(3)	Lien et al., 2020(1); Lien et al., 2020(3)	Lien et al., 2020(1); Lien et al., 2020(2)
Literacy			Howie et al.,2020; Hinkley et al., 2020R; Hinkley et al., 2020S; Hinkley et al., 2020W; Hinkley et al., 2020L			Watson et al., 2022(1)	Watson et al., 2022(1)
Numeracy			Howie et al.,2020; Hinkley et al., 2020	Watson et al., 2022(1)	Watson et al., 2022(1)	Watson et al., 2022(1)	Watson et al., 2022(1)

### Quality assessment of evidence

Due to the inclusion of cross-sectional studies, the level of evidence is low for all. The results of the systematic review do not show obvious limitations, imprecision, indirectness, and publication bias, but there is still significant inconsistency in the results of most outcome variables. Moreover, this study is a systematic review rather than a meta-analysis, so it is unclear whether the results of the combined effect test are due to a larger effect size. The included studies explored the relationship between meeting different combinations of guidelines and not meeting the guidelines. However, in the studies on variables such as inhibitory function and academic performance, some included studies have not strictly controlled the relevant confounding factors, reducing the quality of the evidence.

Studies that meet the PA guidelines show no significant inconsistency in the relationship with most outcome variables, but the results all suggest that the association between meeting the PA guidelines and most outcome variables is not significant. Studies on the relationship between meeting the ST guidelines and fluid intelligence, literacy, and numeracy show no significant inconsistency in the results, but only the association between meeting the ST guidelines and fluid intelligence is significant. Studies on the relationship between meeting the SD guidelines and fluid intelligence, global cognition, inhibitory function, gray matter volume, and literacy show no significant inconsistency in the results, but only the association between meeting the SD guidelines and literacy is significant. Studies on the relationship between meeting both PA and ST guidelines and global cognition, crystallized intelligence, fluid intelligence, gray matter volume, academic performance, and literacy show no significant inconsistency in the results, but the results all suggest that the association between meeting the ST guidelines and the aforementioned variables is not significant. Studies on the relationship between meeting both PA and SD guidelines and global cognition, crystallized intelligence, fluid intelligence, gray matter volume, and literacy show no significant inconsistency in the results, but the results all suggest that the association between meeting the ST guidelines and the aforementioned variables is not significant. Studies on the relationship between meeting both ST and SD guidelines and fluid intelligence, inhibitory function, and gray matter volume show no significant inconsistency in the results, and the associations between meeting both ST and SD guidelines and fluid intelligence and gray matter volume are significant. Studies on the relationship between meeting all guidelines and fluid intelligence and inhibitory function show no significant inconsistency, and the association between meeting all guidelines and fluid intelligence is significant. For detailed results, please refer to Supplementary Material 3.

## Discussion

### Meeting ST guidelines is positively correlated with cognitive function

The findings of this study indicate a positive correlation between adherence to the ST guidelines and cognitive performance, particularly reflected in fluid intelligence. Previous study [53] has confirmed that chronic sensory stimulation due to excessive screen time may have a negative impact on cognitive performance. For instance, Sauce et al. [54] found a significant negative correlation between screen time and children's intellectual development, controlling for genetic differences in education and socioeconomic status. Fikkers et al. [55] discovered an association between children's exposure to video games and fluid intelligence, while the association with crystallized intelligence was not significant. Additionally, studies on mobile phone dependency [56] and internet addiction [57] also support this finding.

The underlying mechanisms for how excessive ST impairs cognitive performance can be explained from several perspectives. Firstly, excessive ST can lead to symptoms such as dry eyes, fatigue, and vision decline [58], which not only affect visual comfort but may also interfere with the normal transmission and processing of visual information [59, 60]. Secondly, the visual cortex is widely connected to other brain regions such as the frontal, parietal, and temporal lobes, which jointly participate in cognitive processes like attention, memory, and decision-making [61]. If visual information is impaired due to screen damage, it will inevitably have adverse effects on brain network connectivity [62]. Finally, the size of the screen and potential blurred or distorted information can cause difficulties for the brain to interpret, thereby increasing cognitive load [63]. Furthermore, exposure to blue light from screens can interfere with the secretion of melatonin, leading to difficulties falling asleep, decreased sleep quality, and subsequent indirect impairment of cognitive performance during the day [64].

The association between adherence to the ST guidelines and the relationship between gray matter volume and academic performance is unclear. The focus of 24-h movement behaviors research on ST is limited to overall ST and does not consider the content and tasks involved in screen exposure. However, the content and tasks of screen exposure may moderate the relationship between the two. For instance, passive television viewing does not require interaction from the information receiver, whereas activities like surfing the internet or playing games require the receiver to control information and respond, which may have inconsistent effects on brain development [65]. Furthermore, moderate-intensity screen media task processing has been found to promote brain plasticity and cognitive development compared to vigorous- or low-intensity task processing [66]. Additionally, if children and adolescents use screens such as phones, tablets, and computers for beneficial learning and cognitive activities, it may not have negative effects on academic performance [67, 68]. Conversely, excessive screen-based entertainment activities may alter individual gray matter integrity [67, 68], which in turn could impair academic performance. Therefore, future studies should consider not only the overall ST but also the specific content and tasks involved in screen exposure to gain a more comprehensive understanding of its impact on brain development and academic performance.

### Meeting SD guidelines is positively correlated with academic performance

The findings of this study indicate that there is a positive correlation between adherence to the SD guidelines and academic performance, with no inconsistency in the association with literacy, a result that has been widely confirmed by previous studies [69, 70]. Conversely, insufficient sleep has been linked to poorer academic performance among students [71, 72]. The underlying mechanisms explaining the relationship between sleep and academic performance can be explained from the perspectives of neurophysiology, mental health, and physical health. Firstly, from a neurophysiological perspective, sleep is a crucial time for the brain to consolidate memories and organize information [73]. Therefore, when students obtain sufficient sleep, their memory and learning efficiency are enhanced, which in turn contributes to improved academic performance. Secondly, in terms of mental health, sleep deprivation can increase the risk of depression and anxiety [74, 75], which can interfere with attention and learning efficiency. Conversely, adequate sleep helps restore emotional balance and enhances motivation for learning. Lastly, from a physical health perspective, maintaining healthy sleep habits is beneficial for growth and development, weight control, and immune function [76–78]. These improvements directly or indirectly have a positive impact on academic performance.

Despite the positive association between gray matter volume, cognitive function, and academic performance [79–81], this study has not yet found a definitive link between meeting the SD guidelines and gray matter volume and cognitive performance. This inconsistency could be attributed to several factors. Firstly, the assessment of cognitive performance involves multiple dimensions such as attention, memory, thinking, and problem-solving, and the beneficial effects of sleep on cognition may vary from person to person. Therefore, even if adhering to SD guidelines promotes academic performance, it may not always yield consistent results when it comes to specific dimensions of cognitive performance. Secondly, factors such as the learning environment and emotional state may, to a certain extent, mask or weaken the positive association between SD and gray matter volume and cognitive performance. It is worth noting that although the association between adherence to the SD guidelines and gray matter volume and cognitive performance is not clear in the included studies, maintaining good sleep is still crucial for the normal functioning of the brain and the improvement of cognitive performance [82, 83]. Future studies should further explore the complex relationship between sleep, brain structure, and cognitive functions to provide more comprehensive insights into the role of sleep in academic and cognitive outcomes.

### Meeting ST and SD guidelines is positively correlated with cognition function and gray matter volume

The findings of this study reveal a positive association between adherence to ST and SD guidelines and cognitive function (especially fluid intelligence), as well as gray matter volume, further supporting the independent effects of the ST and SD guidelines. Moreover, the findings of this study also reinforce previous studies [84, 85] exploring the combined effects of ST and SD. For instance, Dutta et al. [84] demonstrated a correlation between reduced ST and optimal SD with better cognitive performance in children. In addition, Pérez-Chada et al. [86] showed that excessive ST may lead to SD deficiency and daytime sleepiness, which is not conducive to the positive development of brain plasticity, and thus affects the academic performance of children and adolescents. Given that the underlying mechanisms of the positive benefits of ST and SD have been previously discussed, we will not delve into them further in this study.

Furthermore, our study demonstrates a significant positive association between adherence to both ST and SD guidelines and a wider range of outcome variables, suggesting that the combined effects of ST and SD are superior to their individual effects. Multiple original studies have supported this finding. For instance, Walsh et al. [47] discovered a correlation between adhering to both ST and SD guidelines and better global cognitive function, which was not observed when only the SD guidelines were met. Zeng et al. [34] found a similar association between compliance with both ST and SD guidelines and improved performance on the WCST task, but this was not the case when only the ST guidelines were adhered to. Additionally, Fung et al. [48] demonstrated a link between adhering to both ST and SD guidelines and better GMVs, while no such association was observed when only the SD guidelines were followed. In summary, ST and SD have a combined effect on cognition function and gray matter volume in children and adolescents, and this combined effect is superior to the individual effects of ST and SD alone.

### Meeting all guidelines is positively correlated with cognition function

The findings of this study reveal a positive link between adherence to PA, ST, and SD guidelines and cognitive function, especially as there is no inconsistency in the correlation between meeting all guidelines and fluid intelligence. However, it's noteworthy that the role of meeting the PA guidelines may be relatively limited within the 24-h movement behavior guidelines. Firstly, only two studies [34, 37] showed that meeting the PA guidelines was significantly associated with executive function measured by WCST and overall academic performance. Secondly, meeting the ST and SD guidelines was positively associated with more outcome variables, while meeting all guidelines was only associated with fluid intelligence. Therefore, researchers need to reevaluate the effectiveness and rationality of the PA guidelines from the perspective of cognitive enhancement.

One potential explanation for the relatively lower effect of PA may be that the PA guidelines may not be entirely suitable for cognitive-related outcomes. The PA guidelines recommend at least 60 min of MVPA per day for children and adolescents. However, some studies [87, 88] suggests that there is an inverted U-shaped relationship between PA intensity and cognitive performance, indicating that moderate PA is most effective in enhancing cognitive function, while vigorous PA may impair it. Additionally, the strength model of self-control suggests that self-control resources are limited. If the energy expended by previous self-control tasks is not promptly restored, it may lead to self-depletion, which can affect subsequent self-control task performance [89]. Furthermore, the promotion of cognitive performance may be related to the type of activity, with tasks that emphasize flexibility and coordination being more beneficial for enhancing cognitive performance [90]. However, the PA guidelines do not take into account the type of activity as a factor. Based on these considerations, this study calls for a more comprehensive approach when developing PA guidelines and 24-h movement behavior guidelines. Factors such as the intensity and type of PA should be taken into account to ensure the scientific validity and effectiveness of the guidelines.

### Limitations of this study

There are still some limitations in this study. Firstly, given the differences in statistical techniques and confounding factors adopted in the original studies, it is difficult to extract the effect sizes for a combined effect test. Therefore, this study adopts the idea of frequency analysis to investigate the degree of consistency in the results of the included studies. However, this imprecise statistical method still needs further testing in subsequent studies. Secondly, the quality of the included original studies needs to be improved, especially in the selection strategy and criteria for subjects, the reliability and validity of data collection tools, and the measures to verify the authenticity of the data. Secondly, due to the limited number of original studies, this study did not conduct further analysis on other factors such as age, gender, and ethnicity. Therefore, it is still unclear about the heterogeneity of the research results across different groups.

## Conclusion

This study conducted a systematic review to explore the relationship between adherence to the 24-h movement behavior guidelines and the cognitive function, gray matter volume, and academic performance of children and adolescents. The included studies generally agreed upon the following associations: adherence to the ST guidelines is positively linked to fluid intelligence; adherence to the SD guidelines is positively linked to literacy; adherence to both ST and SD guidelines is associated with increased fluid intelligence and gray matter volume; and overall adherence to all guidelines is positively correlated with fluid intelligence. These findings enrich the research in the field of 24-h movement behaviors and provide a basis for policy formulation aimed at improving the cognitive and academic performance of children and adolescents. Moreover, the role of adhering to PA guidelines in the 24-h movement behavior guidelines may be relatively limited, and vigorous PA may even impair cognitive performance. Therefore, researchers need to reconsider the effectiveness and rationality of PA guidelines and 24-h movement behavior guidelines from the perspective of cognitive enhancement. Future studies should focus on the role of PA intensity and type in cognitive performance to further improve PA guidelines and 24-h movement behavior guidelines.

## Supplementary Information

Supplementary Material 1.

Supplementary Material 2.

Supplementary Material 3.

## Data Availability

The datasets used and/or analyzed during the current study available from the corresponding author on reasonable request.
