# Supplementary material for: Associations between meeting 24h movement behavior guidelines and cognition, gray matter volume, and academic performance in children and adolescents: a systematic review
Source: Arch Public Health. 2025 Jan 10;83:10. doi: 10.1186/s13690-024-01493-0 (PMC11720839; doi:10.1186/s13690-024-01493-0)
Supplement: Supplementary file 2 — Supplementary Material 2. [file 13690_2024_1493_MOESM2_ESM.docx]

# Supplementary Material 2: Literature search process and results

(1) CNKI: SU = ('儿童' + '青少年') * ('24h' + '24h活动' + '24小时') * ('认知' + '执行功能' + '注意' + '自我控制' + '自我调节' + '灰质' + '学业表现' + '学习成绩') [in Chinese]

After the search, a total of 26 articles were retrieved in this study.

(2) WOS: TS = ((children OR adolescent OR youth OR teenager OR preschool OR juvenile OR pupil OR primary OR elementary OR high school OR junior school OR senior school) AND (24-hour OR 24-hour movement) AND (cognitive OR executive function OR attention OR self-control OR gray matter OR academic performance))

After the search, a total of 508 articles were retrieved in this study.

(3) PubMed: TS = ((children OR adolescent OR youth OR teenager OR preschool OR juvenile OR pupil OR primary OR elementary OR high school OR junior school OR senior school) AND (24-hour OR 24-hour movement) AND (cognitive OR executive function OR attention OR self-control OR gray matter OR academic performance))

After the search, a total of 871 articles were retrieved in this study.

(4) SPORT Discus: TX = ((children OR adolescent OR youth OR teenager OR preschool OR juvenile OR pupil OR primary OR elementary OR high school OR junior school OR senior school) AND (24-hour OR 24-hour movement) AND (cognitive OR executive function OR attention OR self-control OR gray matter OR academic performance))

After the search, a total of 155 articles were retrieved in this study.

(5) Cochrane Library: ((children OR adolescent OR youth OR teenager OR preschool OR juvenile OR pupil OR primary OR elementary OR high school OR junior school OR senior school) AND (24 hour OR 24 hour movement) AND (cognitive OR executive function OR attention OR self-control OR gray matter OR academic performance)) in Title Abstract Keyword - (Word variations have been searched)

After the search, a total of 2638 articles were retrieved in this study.
