# Supplementary material for: Associations between meeting 24h movement behavior guidelines and cognition, gray matter volume, and academic performance in children and adolescents: a systematic review
Source: Arch Public Health. 2025 Jan 10;83:10. doi: 10.1186/s13690-024-01493-0 (PMC11720839; doi:10.1186/s13690-024-01493-0)
Supplement: Supplementary file 3 — Supplementary Material 3. [file 13690_2024_1493_MOESM3_ESM.docx]

# Supplementary Material 3: A summary of evidence on the association between meeting the 24h movement behavior guidelines and cognition, gray matter volume, and academic performance in children and adolescents

| **Variables** | **Guidelines** | **Downgrade** | | | | | **Upgrade** | | | **Quality** |
| --- | --- | --- | --- | --- | --- | --- | --- | --- | --- | --- |
|  |  | **Limitation** | **Imprecision** | **Inconsistency** | **Indirect** | **Publication bias** | **Large effect size** | **Dose-response** | **Minimizing confounding factors** |  |
| Crystallized intelligence | PA | No serious limitations | No serious imprecision | No serious inconsistency | No serious indirect | No found | Unclear | Yes | Strict control of confounding factors | Low |
|  | ST | No serious limitations | No serious imprecision | 1/3 of the studies did not show positive results | No serious indirect | No found | Unclear | Yes | Strict control of confounding factors | Low |
|  | SD | No serious limitations | No serious imprecision | 1/3 of the studies did not show positive results | No serious indirect | No found | Unclear | Yes | Strict control of confounding factors | Low |
|  | PA+ST | No serious limitations | No serious imprecision | No serious inconsistency | No serious indirect | No found | Unclear | Yes | Strict control of confounding factors | Low |
|  | PA+SD | No serious limitations | No serious imprecision | No serious inconsistency | No serious indirect | No found | Unclear | Yes | Strict control of confounding factors | Low |
|  | ST+SD | No serious limitations | No serious imprecision | 1/3 of the studies did not show positive results | No serious indirect | No found | Unclear | Yes | Strict control of confounding factors | Low |
|  | PA+ST+SD | No serious limitations | No serious imprecision | 1/3 of the studies did not show positive results | No serious indirect | No found | Unclear | Yes | Strict control of confounding factors | Low |
| Fluid intelligence | PA | No serious limitations | No serious imprecision | No serious inconsistency | No serious indirect | No found | Unclear | Yes | Strict control of confounding factors | Low |
|  | ST | No serious limitations | No serious imprecision | No serious inconsistency | No serious indirect | No found | Unclear | Yes | Strict control of confounding factors | Low |
|  | SD | No serious limitations | No serious imprecision | No serious inconsistency | No serious indirect | No found | Unclear | Yes | Strict control of confounding factors | Low |
|  | PA+ST | No serious limitations | No serious imprecision | No serious inconsistency | No serious indirect | No found | Unclear | Yes | Strict control of confounding factors | Low |
|  | PA+SD | No serious limitations | No serious imprecision | No serious inconsistency | No serious indirect | No found | Unclear | Yes | Strict control of confounding factors | Low |
|  | ST+SD | No serious limitations | No serious imprecision | No serious inconsistency | No serious indirect | No found | Unclear | Yes | Strict control of confounding factors | Low |
|  | PA+ST+SD | No serious limitations | No serious imprecision | No serious inconsistency | No serious indirect | No found | Unclear | Yes | Strict control of confounding factors | Low |
| Global cognition | PA | No serious limitations | No serious imprecision | No serious inconsistency | No serious indirect | No found | Unclear | Yes | Strict control of confounding factors | Low |
|  | ST | No serious limitations | No serious imprecision | 1/3 of the studies did not show positive results | No serious indirect | No found | Unclear | Yes | Strict control of confounding factors | Low |
|  | SD | No serious limitations | No serious imprecision | No serious inconsistency | No serious indirect | No found | Unclear | Yes | Strict control of confounding factors | Low |
|  | PA+ST | No serious limitations | No serious imprecision | No serious inconsistency | No serious indirect | No found | Unclear | Yes | Strict control of confounding factors | Low |
|  | PA+SD | No serious limitations | No serious imprecision | No serious inconsistency | No serious indirect | No found | Unclear | Yes | Strict control of confounding factors | Low |
|  | ST+SD | No serious limitations | No serious imprecision | 1/3 of the studies did not show positive results | No serious indirect | No found | Unclear | Yes | Strict control of confounding factors | Low |
|  | PA+ST+SD | No serious limitations | No serious imprecision | 1/3 of the studies did not show positive results | No serious indirect | No found | Unclear | Yes | Strict control of confounding factors | Low |
| Inhibitory function | PA | No serious limitations | The total sample size is too small | No serious inconsistency | No serious indirect | No found | Unclear | Yes | Confounding factors were not strictly controlled | Low |
|  | ST | No serious limitations | The total sample size is too small | Half of the studies did not show positive results | No serious indirect | No found | Unclear | Yes | Confounding factors were not strictly controlled | Low |
|  | SD | No serious limitations | The total sample size is too small | No serious inconsistency | No serious indirect | No found | Unclear | Yes | Confounding factors were not strictly controlled | Low |
|  | PA+ST | No serious limitations | The total sample size is too small | Half of the studies did not show positive results | No serious indirect | No found | Unclear | Yes | Confounding factors were not strictly controlled | Low |
|  | PA+SD | No serious limitations | The total sample size is too small | Half of the studies did not show positive results | No serious indirect | No found | Unclear | Yes | Confounding factors were not strictly controlled | Low |
|  | ST+SD | No serious limitations | The total sample size is too small | No serious inconsistency | No serious indirect | No found | Unclear | Yes | Confounding factors were not strictly controlled | Low |
|  | PA+ST+SD | No serious limitations | The total sample size is too small | No serious inconsistency | No serious indirect | No found | Unclear | Yes | Confounding factors were not strictly controlled | Low |
| GMVs | PA | No serious limitations | No serious imprecision | No serious inconsistency | No serious indirect | No found | Unclear | Yes | Strict control of confounding factors | Low |
|  | ST | No serious limitations | No serious imprecision | Half of the studies did not show positive results | No serious indirect | No found | Unclear | Yes | Strict control of confounding factors | Low |
|  | SD | No serious limitations | No serious imprecision | No serious inconsistency | No serious indirect | No found | Unclear | Yes | Strict control of confounding factors | Low |
|  | PA+ST | No serious limitations | No serious imprecision | No serious inconsistency | No serious indirect | No found | Unclear | Yes | Strict control of confounding factors | Low |
|  | PA+SD | No serious limitations | No serious imprecision | No serious inconsistency | No serious indirect | No found | Unclear | Yes | Strict control of confounding factors | Low |
|  | ST+SD | No serious limitations | No serious imprecision | No serious inconsistency | No serious indirect | No found | Unclear | Yes | Strict control of confounding factors | Low |
|  | PA+ST+SD | No serious limitations | No serious imprecision | Half of the studies did not show positive results | No serious indirect | No found | Unclear | Yes | Strict control of confounding factors | Low |
| Overall academic performance | PA | No serious limitations | No serious imprecision | 6/7 of the studies did not show positive results | No serious indirect | No found | Unclear | Yes | Some studies have not yet strictly controlled for confounding factors | Low |
|  | ST | No serious limitations | No serious imprecision | 6/7 of the studies did not show positive results | No serious indirect | No found | Unclear | Yes | Some studies have not yet strictly controlled for confounding factors | Low |
|  | SD | No serious limitations | No serious imprecision | 2/7 of the studies did not show positive results | No serious indirect | No found | Unclear | Yes | Some studies have not yet strictly controlled for confounding factors | Low |
|  | PA+ST | No serious limitations | No serious imprecision | No serious inconsistency | No serious indirect | No found | Unclear | Yes | Some studies have not yet strictly controlled for confounding factors | Low |
|  | PA+SD | No serious limitations | No serious imprecision | Half of the studies did not show positive results | No serious indirect | No found | Unclear | Yes | Some studies have not yet strictly controlled for confounding factors | Low |
|  | ST+SD | No serious limitations | No serious imprecision | 1/3 of the studies did not show positive results | No serious indirect | No found | Unclear | Yes | Some studies have not yet strictly controlled for confounding factors | Low |
|  | PA+ST+SD | No serious limitations | No serious imprecision | 1/3 of the studies did not show positive results | No serious indirect | No found | Unclear | Yes | Some studies have not yet strictly controlled for confounding factors | Low |
| Literacy | PA | No serious limitations | No serious imprecision | No serious inconsistency | No serious indirect | No found | Unclear | Yes | Some studies have not yet strictly controlled for confounding factors | Low |
|  | ST | No serious limitations | No serious imprecision | No serious inconsistency | No serious indirect | No found | Unclear | Yes | Some studies have not yet strictly controlled for confounding factors | Low |
|  | SD | No serious limitations | No serious imprecision | No serious inconsistency | No serious indirect | No found | Unclear | Yes | Some studies have not yet strictly controlled for confounding factors | Low |
|  | PA+ST | No serious limitations | No serious imprecision | No serious inconsistency | No serious indirect | No found | Unclear | Yes | Strict control of confounding factors | Low |
|  | PA+SD | No serious limitations | No serious imprecision | No serious inconsistency | No serious indirect | No found | Unclear | Yes | Strict control of confounding factors | Low |
|  | ST+SD | No serious limitations | No serious imprecision | Half of the studies did not show positive results | No serious indirect | No found | Unclear | Yes | Strict control of confounding factors | Low |
|  | PA+ST+SD | No serious limitations | No serious imprecision | Half of the studies did not show positive results | No serious indirect | No found | Unclear | Yes | Strict control of confounding factors | Low |
| Numeracy | PA | No serious limitations | No serious imprecision | No serious inconsistency | No serious indirect | No found | Unclear | Yes | Some studies have not yet strictly controlled for confounding factors | Low |
|  | ST | No serious limitations | No serious imprecision | No serious inconsistency | No serious indirect | No found | Unclear | Yes | Some studies have not yet strictly controlled for confounding factors | Low |
|  | SD | No serious limitations | No serious imprecision | Half of the studies did not show positive results | No serious indirect | No found | Unclear | Yes | Some studies have not yet strictly controlled for confounding factors | Low |
|  | PA+ST | No serious limitations | No serious imprecision | Half of the studies did not show positive results | No serious indirect | No found | Unclear | Yes | Strict control of confounding factors | Low |
|  | PA+SD | No serious limitations | No serious imprecision | Half of the studies did not show positive results | No serious indirect | No found | Unclear | Yes | Strict control of confounding factors | Low |
|  | ST+SD | No serious limitations | No serious imprecision | Half of the studies did not show positive results | No serious indirect | No found | Unclear | Yes | Strict control of confounding factors | Low |
|  | PA+ST+SD | No serious limitations | No serious imprecision | Half of the studies did not show positive results | No serious indirect | No found | Unclear | Yes | Strict control of confounding factors | Low |
